# A strong diffusive ion mode in dense ionized matter predicted by Langevin dynamics

**DOI:** 10.1038/ncomms14125

**Published:** 2017-01-30

**Authors:** P. Mabey, S. Richardson, T. G. White, L. B. Fletcher, S. H. Glenzer, N. J. Hartley, J. Vorberger, D. O. Gericke, G. Gregori

**Affiliations:** 1 Department of Physics, University of Oxford, Parks Road, Oxford OX1 3PU, UK; 2 AWE, Aldermaston, Reading, Berkshire RG7 4PR, UK; 3 SLAC National Accelerator Laboratory, 2575 Sand Hill Road, MS 19, Menlo Park, California 94025, USA; 4 Physics Department, University of California Berkeley, Berkeley, California 94709, USA; 5 Institute of Radiation Physics, Helmholtz-Zentrum Dresden-Rossendorf, 01328 Dresden, Germany; 6 Centre for Fusion, Space and Astrophysics, Department of Physics, University of Warwick, Coventry CV4 7AL, UK

## Abstract

The state and evolution of planets, brown dwarfs and neutron star crusts is determined by the properties of dense and compressed matter. Due to the inherent difficulties in modelling strongly coupled plasmas, however, current predictions of transport coefficients differ by orders of magnitude. Collective modes are a prominent feature, whose spectra may serve as an important tool to validate theoretical predictions for dense matter. With recent advances in free electron laser technology, X-rays with small enough bandwidth have become available, allowing the investigation of the low-frequency ion modes in dense matter. Here, we present numerical predictions for these ion modes and demonstrate significant changes to their strength and dispersion if dissipative processes are included by Langevin dynamics. Notably, a strong diffusive mode around zero frequency arises, which is not present, or much weaker, in standard simulations. Our results have profound consequences in the interpretation of transport coefficients in dense plasmas.

## Abstract (web summary)

Studying the properties of dense plasmas is challenging due to strong interactions between electrons and ions, and numerical methods overcome this difficulty using a static thermostat. Here the authors predict a strong diffusive ion mode at low energy by including dissipative processes in the model.

The Langevin approach was introduced to describe, in a stochastic manner, damping in the one-particle dynamics originating from omitted degrees of freedom. A typical example is Brownian motion where the random weak collisions with the background gas or fluid usually cannot be treated explicitly. The resulting diffusive process creates a zero-frequency mode and its strength may thus serve as an estimate of the effects of randomization processes. This approach has previously been successfully implemented in the field of dusty plasmas to model the effects of neutral species on the diffusion coefficient^1^,^2^,^3^. In the case of dense ionized matter, Langevin dynamics may mimic processes like dynamic electron–ion collisions^4^ usually not included in standard simulations.

Dense matter with moderate temperatures determines the state and evolution of many astrophysical objects such as giant gas planets, brown and white dwarf stars and the crust of neutron stars^5^,^6^,^7^,^8^. Moreover, similar conditions occur at several stages of implosions aiming to achieve inertial confinement fusion^9^,^10^, as well as during material processing^11^. Laboratory experiments are now able to create these conditions with a range of techniques allowing critical tests of theory and modelling^12^,^13^,^14^,^15^,^16^. Still, the experimental possibilities to diagnose dense matter are rather limited and a thorough comparison of simulations and data is often the only way to reveal the microscopic behaviour.

Modelling matter in this parameter regime is very challenging as one often encounters systems with strong interactions as well as electrons that exhibit distinct quantum behaviour. Thus, either first-principle simulations or reduced models are applied to determine the system properties. Investigating the ion dynamics is particularly challenging as the pure Coulomb interactions between the ions are modified by electron structure, that is, the bound state properties and screening^17^,^18^. In turn, this behaviour makes the ion modes very interesting as they encode almost the entire system behaviour including electron properties^19^.

In the last few years, the ion modes have been investigated using simulations with increasing complexity: molecular dynamics (MD) simulations using model potentials^20^ and potentials extracted from *ab initio* simulations^21^, orbital-free density functional theory (OF-DFT)^22^, and most recently full Kohn–Sham density functional theory (KS-DFT)^23^ have been applied. Such simulations are typically run with constant particle number, volume and temperature (NVT ensemble) and, thus, require a numerical thermostat to keep the ionic temperature constant. Moreover, while some efforts have been made to include electron–ion relaxation with classical MD at very high temperatures^24^,^25^, DFT-MD simulations required for coupled electron–ion systems in the warm dense matter (WDM) regime always use the Born–Oppenheimer approximation, neglecting the dynamics of the electron–ion interactions.

In our simulations, we investigate the effect of the form of the thermostat on the dynamic structure factor of the ions. We show that this quantity reveals critical information on events randomizing the ion dynamics. Our modelling is based on the Langevin approach, which contains a free parameter that encodes the strength of the damping of the modes. We compare our results with the often-used Nosé–Hoover and Gaussian thermostats within the framework of OF-DFT MD, as well as classical MD simulations. Our simulation results are motivated by recent experimental data showing strong scattering around zero frequency^26^, suggesting that the dynamic electron–ion effects in this regime do indeed have a crucial role in the ion dynamics and hence conventional thermostats do not adequately describe the system.

## Results

### Simulations with different thermostats

We have performed OF-DFT simulations coupled to classical MD for the ions as well as purely classical MD simulations. For the ions, we solve the equations of motion


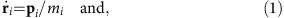



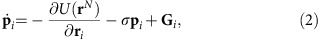


where *U* denotes the interaction potential between the ions. Within the OF-DFT scheme, this potential is self-consistently calculated from the electron densities and the basic Coulomb interaction of the ions; within classical MD, it is an effective ion–ion interaction usually taking linear screening of the Coulomb forces into account. More advanced models consider an additional short-range repulsion^27^,^28^. The second term is the usual thermostat that sets a specific temperature in the ion system by re-scaling the momenta, where *σ* controls the time scale of reaching the required temperature. Often applied thermostats, like the isokinetic (Gaussian)^29^ or the Nosé–Hoover^30^,^31^ descriptions, contain only this term (**G**_*i*_=0, see the ‘Methods' section for a full definition).

Within the Langevin dynamics, the additional third term, **G**_*i*_, describes a Gaussian random force randomizing the one-particle dynamics. This force is set to have a zero average and variance of 2*σk*_B_*T*. The magnitude of the random force applied to the ions is set using the fluctuation–dissipation relation such that the ions follow a Maxwell–Boltzmann distribution with a specified temperature. Thus, the parameters *σ* and **G**_*i*_ are connected and there is only one free parameter as in the other schemes. The strict mathematical range of validity of the Langevin approach is not trivial to determine (see, for example, ref. 32 for details). As it is applied here, one requires that the plasma behaves as a liquid^33^ and that the value of *σ* is chosen such that the temperature and pressure of the system remain constant during the simulation^34^. Both are well fulfilled for the example shown.

### Dynamic ion–ion structure factor

The dynamic structure factor (DSF) contains the dynamics of the particles and the related collective excitations. It is closely connected to the dielectric response via the fluctuation–dissipation theorem and, in principle, all other statistical and thermodynamic properties can be derived from the DSF^35^. It also provides an important connection to experiments allowing for thorough tests of theoretical models and simulations of WDM^36^,^37^ as it is proportional to the X-ray scattering cross-section^38^.

The DSF describes density fluctuations with frequency *ω* and wavenumber **k** and, for systems in thermodynamic equilibrium, is defined as


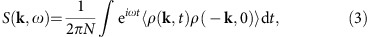


where *N* is the number of particles and the brackets <...> represent an ensemble average. For the ion–ion structure factor considered here, the quantity *ρ* is the Fourier transform of the time-dependent ion density in real space: 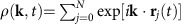.

The DSF exhibits the collective modes as pronounced peaks. For the ions, one typically finds three modes: a diffusive mode centred around zero frequency and two ion acoustic modes whose positions are defined by the sound velocity. The latter are the ionic equivalent of collective electron plasma waves (plasmons), which have already been observed in WDM^15^,^39^,^40^,^41^. Although the separation of the ion acoustic modes is much smaller, they can be resolved with radiation of extremely low bandwidth as provided by free electron lasers^42^.

### The mode structure in warm dense matter

Here, we investigate the relative strength of the diffusive and acoustic modes in the ion–ion structure factor with OF-DFT simulations using the three different thermostats as described above. Tests against simulations applying KS-DFT have shown that the efficient OF-DFT yields excellent agreement for the examples studied here^22^. To illustrate effects purely related to the thermostat, we also include results from fully classical MD simulations using a Yukawa potential with a short-range repulsion term^22^. As an example, we consider heated aluminium compressed to twice the solid density: *T*=3.5 eV and *ρ*=5.2g cm^−3^.

**Figure 1:**
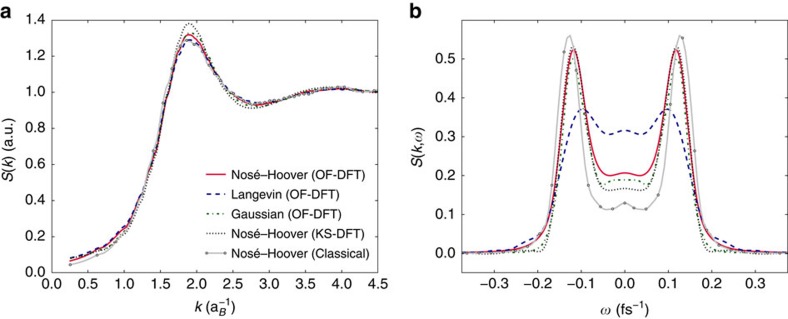
Static and dynamic structure factors of warm dense aluminium. (**a**) The ion–ion static structure factor. (**b**) The ion–ion dynamic structure factor at *k*=0.51 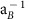. The structure factors (*T*_*e*_=*T*_*i*_=3.5 eV and *ρ*=5.2 g cm^−3^), are calculated from orbital-free density functional theory (OF-DFT) simulations in a canonical ensemble with a Nosé–Hoover, Langevin and Gaussian thermostat; The Langevin thermostat uses a collision induced friction, *σ*, of 6 × 10^13^ s^−1^. For comparison, results from a Kohn–Sham density functional theory (KS-DFT) simulation and a fully classical simulation using a screened Coulomb potential with an added short-range repulsion, both in a canonical ensemble with a Nosé–Hoover thermostat (dotted) are also included.

For the conditions above, all simulations, OF-DFT as well as classical MD, yield very similar static structure factors, SSF, (frequency integrated DSF) regardless of the thermostats used, as shown by Fig. 1a. Thus, static properties, including the equation of states being derived from it, are insensitive to the choice of the thermostat justifying the standard simulations usually using a Nosé–Hoover approach. This is to be expected: static properties of plasmas in equilibrium, such as the SSF, should indeed be insensitive to the choice of thermostat and the kinetic coefficients. Figure 1b demonstrates that the DSF is, on the contrary, very sensitive to the choice made for the thermostat. Although the results calculated with the Nosé–Hoover and Gaussian thermostats exhibit little difference, the DSF from the Langevin dynamics clearly shows a different mode structure: the two peaks representing the ion acoustic modes, symmetric around the origin, are strongly damped when the additional damping in the Langevin formalism is introduced. Moreover, the central part around zero frequency is strongly enhanced. This central feature is often referred to as the Rayleigh line and arises from entropy (temperature) fluctuations at constant pressure^43^. The occurrence of this diffusive mode is directly caused by the inclusion of the random force term, **G**_*i*_, in the Langevin approach.

It is obvious that selecting the correct value of *σ* is crucial to obtain accurate results. For a first test, we have used a value of *σ*=6 × 10^13^ s^−1^ from ref. 4, that has been calculated using the Rayleigh model^44^. We have also used a value of *σ*=1 × 10^14^ s^−1^ calculated within the Born approximation with a statically screened Coulomb potential^45^. Additional examples are simulated to investigate the sensitivity of the mode structure to the parameter *σ*. Ultimately, only experiments will be able to tell us which is the correct value of *σ* to be used.

**Figure 2:**
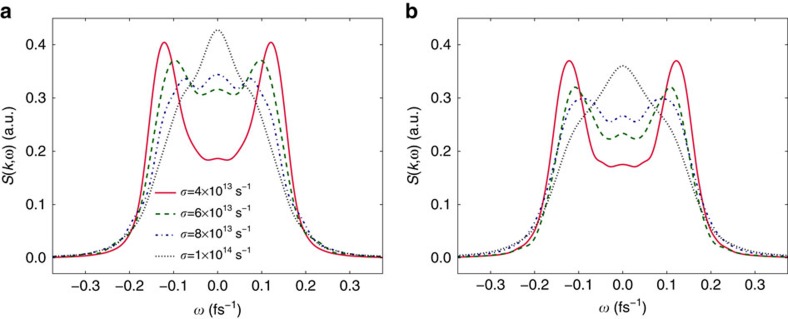
Sensitivity of the dynamic ion–ion structure factor on the Langevin friction parameter *σ*. Data were obtained from orbital-free density functional theory (OF-DFT) simulations (**a**) and fully classical MD simulations (**b**) for warm dense aluminium at *T*_*e*_=*T*_*i*_=3.5 eV and *ρ*=5.2 g cm^−3^. The classical and orbital-free approaches both exhibit the same trend; that is, the central Rayleigh line dominates the acoustic peaks at the largest value of *σ* considered, whereas the central peak disappears altogether at lower values.

Figure 2 demonstrates that the central Rayleigh line dominates the acoustic peaks at the largest *σ* considered, whereas the central peak disappears altogether at lower values. In the latter case, the DSF simply reduces to that produced by either of the conventional thermostats, thus suggesting that the effects of electron–ion dynamics are negligible in this scenario. The different considered values of *σ* here span the transition region from a highly diffusive system to one dominated by acoustic modes.

**Figure 3:**
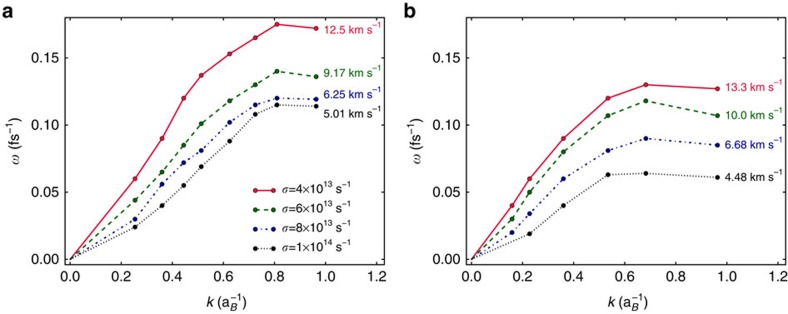
Dispersion relations of the ion acoustic modes for warm dense aluminium with varying friction strength. (**a**) Shows data from orbital-free density functional theory (OF-DFT) simulations, whereas (**b**) shows data from fully classical MD simulations. Both were run in the canonical ensemble at *T*_*e*_=*T*_*i*_=3.5 eV and *ρ*=5.2 g cm^−3^, using a Langevin thermostat with different friction strengths, *σ*. The annotated numbers give the sound speeds of the ion acoustic waves in the system as obtained from the constant gradient at small wavenumbers, *k*.

We also note that the sound speed of the system is significantly modified by adding the random friction force within the Langevin dynamics. Figure 3 displays the respective dispersion relations for the acoustic peaks explicitly. Moreover, it shows that this trend can be found in both quantum simulations of coupled electron–ion systems and classical simulations of the effective ionic systems. It should be noted at this stage, that the corresponding SSFs for each friction force remain the same, as expected.

It is apparent that the simulations are very sensitive to the choice of *σ* within the range of predicted values. Thus, taking the correct value is essential to predict the dynamic ion properties. Besides the DSF, large effects can be expected for particle diffusion, and energy transfer between species. Indeed, measurements have found large discrepancies with predictions for the timescale of electron–ion equilibration^46^,^47^,^48^. Moreover, this choice may have important implications for transport properties like the stopping power when being extracted from simulations, which offers another experimental test of the method^49^.

Although a theoretical prediction of the most appropriate value of the ion mode damping rate proves to be relatively difficult, the relation of the DSF to the scattering spectrum of X-ray photons can be used to determine its value. The band width of the photons from free electron lasers can be reduced sufficiently to distinguish between the different ion modes^42^. Our simulation results are supported by recent experimental data showing strong scattering around zero frequency (G. Monaco *et al*., manuscript in preparation) These data suggest that the dynamic damping effects in this regime do indeed have a crucial role in the ion dynamics and hence conventional thermostats do not adequately describe the system. Such data can be used to infer the value of *σ* by considering the relative intensity of the acoustic peaks to the central peak^50^.

The physical meaning of the random force in the Langevin equation is, of course, not uniquely defined. Indeed, this approach has already been implemented in the field of dusty plasmas to mimic the effects of neutral species, showing significant changes in both the diffusion constant^1^,^2^ and the intermediate scattering function^3^. In the case of dense, ionized matter, electron–ion collisions have long been discussed as an additional source of ionic energy fluctuations. This type of matter can be seen as classical ions embedded in the background of a degenerate electron fluid. Examples of implementations are numerous: the authors of refs 51, 52 proposed applying a damping force to the ions to mimic electron–ion interactions in MD simulations, a stochastic force was included to represent the energy fed into the ionic system by the electrons^53^, the authors of ref. 54 noted that electrons could act as a heat sink or a heat bath, depending on the various timescales of the system. Once one identifies the random force with dynamic electron–ion collisions, the changes in the DSF we report here also allow for assessing the strength of such collisions determining many transport and relaxation phenomena, a long standing problem in the WDM regime.

## Discussion

Our results demonstrate the importance of properly including all effects randomizing the ionic motion when considering dynamic properties with MD simulations in the WDM regime. Significant changes arise for systems with strong damping, where a strong diffusive peak can be found in the DSF. Moreover, the strength and dispersion of the ion acoustic peaks change, which in turn is associated with changes in the diffusivity and other transport coefficients. Although these effects, could, to some extent, be predicted by the theory of electromagnetic fluctuations in plasmas^55^,^56^ or by considering other simpler damped oscillatory systems, this work shows that they have been observed in classical MD as well as orbital-free DFT-MD simulations of dense plasmas. Although the Langevin approach has been used in the study of dense plasmas previously, its use in this context is entirely different. By making use of this method, we have clearly demonstrated that standard *ab initio* simulations using the Born–Oppenheimer approximation and the conventional Gaussian or Nosé–Hoover thermostats should only be used to obtain static properties like the equation of state. To assess the dynamics of the ionic system, a proper description of all interactions within the system is required. Our work also illustrates the wealth of information contained in the dynamic ion structure and the difficulties modelling this quantity with the same accuracy and predictive power as known from *ab initio* simulations of static or thermodynamic properties of warm dense matter.

## Methods

### OF-DFT simulations

Our OF-DFT simulations were performed using the *ABINIT* package^57^,^58^,^59^ using the Thomas–Fermi module and the exchange-correlation terms in local density approximation. Simulations were run using a 256-ion cubic supercell with periodic boundary conditions. The local pseudopotential used was checked for accuracy by reproducing electron density within a bulk material (see ref. 60 for details). In addition, the effects of increasing the simulation size from 256 up to 864 ions were studied with no changes observed.

### KS-DFT simulations

For comparison, the Vienna *Ab Initio* Simulation Package^61^,^62^,^63^ was also used to perform a full KS-DFT simulation using the Nosé–Hoover thermostat to verify the accuracy of the results. The parameters of the simulation were identical to the OF-DFT although the simulation size was limited to 256 atoms.

### Classical MD simulations

We also include results from fully classical MD simulations using a Yukawa potential with a short range repulsion term^22^. The parameter *b* is determined by the requirement that the SSF obtained via classical simulations reproduce the KS-DFT SSF^21^ while *κ* is taken as the Debye screening length for the system. The classical simulations were performed in the LAMMPS package^64^ with a total of 2,048 atoms using the Nosé–Hoover thermostat. In all three methods, the system was evolved with a timestep of 0.5 fs and run for 50,000 time steps giving an overall simulation time of 25 ps.

### Comparison of thermostats

One of the simplest commonly used techniques is the Gaussian thermostat (sometimes referred to as the isokinetic ensemble) derived by using Gauss' principle of least constraint. It produces the canonical ensemble in the coordinate part of phase space^29^ by using time-reversible and deterministic equations of motion^65^. For the parameter determining the ion temperature, we have


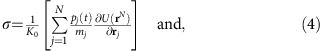



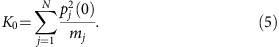


One drawback associated with this thermostat is that conventional ODE solvers will typically exhibit kinetic energy drifting. Thus, one has to introduce a method of *ad hoc* velocity scaling such as that proposed by ref. 66.

Another widely used approach was proposed by Nosé and Hoover^30^,^31^. This Nosé–Hoover thermostat is based on an extended Lagrangian containing additional artificial coordinates and velocities. The equations of motion are the same as for the Gaussian thermostat but with a coefficient set to be


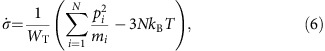


where *W*_T_ is the inertia factor of the thermostat.

An alternative approach is to use the Langevin equation^67^. As discussed, this approach uses an inertial friction coefficient and a Gaussian random force with zero average and variance of 2*σm*_*i*_*k*_B_*T*. Different approaches for the free parameter *σ* are discussed.

### Data availability

The authors declare that the data supporting the findings of this study are available from the authors on request.

## Additional information

**How to cite this article:** Mabey, P. *et al*. A strong diffusive ion mode in dense ionized matter predicted by Langevin dynamics. *Nat. Commun.* **8,** 14125 doi: 10.1038/ncomms14125 (2017).

**Publisher's note**: Springer Nature remains neutral with regard to jurisdictional claims in published maps and institutional affiliations.
